# The development of the primary care general practitioner cluster model: experiences from Hungary

**DOI:** 10.3389/frhs.2026.1769211

**Published:** 2026-03-30

**Authors:** Ferenc Maráczi, Attila Virág, Gergő Túri, Péter Pikó, Csilla Kaposvári, Rita Teller, Csaba László Dózsa, István Vingender

**Affiliations:** 1 Doctoral School of Health Sciences, Faculty of Health Sciences, Semmelweis University, Budapest, Hungary; 2 Faculty of Humanities and Social Sciences, Pázmány Péter Catholic University, Budapest, Hungary; 3 Epidemiology and Surveillance Centre, Semmelweis University, Budapest, Hungary; 4 Synthesis Health Research Foundation, Budapest, Hungary; 5 Department of Public Health and Epidemiology, Faculty of Medicine, University of Debrecen, Debrecen, Hungary; 6 Department of Public Health, Semmelweis University, Budapest, Hungary; 7 Faculty of Health Sciences, Doctoral School, University of Pécs, Pécs, Hungary; 8 Faculty of Humanities, Eötvös Loránd University, Budapest, Hungary; 9 Institute of Theoretical Health Sciences, University of Miskolc, Miskolc, Hungary; 10 Faculty of Health and Sport Sciences, University of Győr, Győr, Hungary

**Keywords:** cluster, dental, general practice, group practice, Hungary, model, network, primary care

## Abstract

**Background:**

In 2021, Hungary introduced general practitioner (GP) clusters to improve access to local healthcare and strengthen cooperation among primary care providers. However, there is limited evidence on how these clusters operate.

**Objective:**

The aim of this research is to provide a comprehensive description of how GP practices and clusters function, and to describe variables associated with a GP's participation in a cluster.

**Methods:**

We analysed national data on general practices and GP clusters in Hungary for the period 2021–2024. This data was obtained from the National Hospital Directorate and supplemented with information from publicly available sources. After harmonising and integrating the data, we used descriptive statistical methods to characterise the operation of practices and clusters. We also used multivariate regression models to examine factors associated with GPs' decisions to join a cluster.

**Results:**

Between 2021 and 2024, the number of GP clusters increased from 365 to 422. Nearly half of all practices had joined by 2024. On average, clusters included seven practices, with one-third collaborating with dentists. Significant regional disparities were observed in cluster participation, while the adoption of digital health technologies and point-of-care tests remained limited. While GP clusters have expanded nationwide, participation appears higher in better-resourced districts.

**Conclusion:**

As in many other developed countries, GP clusters have become increasingly widespread in Hungary. One-third of these clusters involve collaboration between GPs and dentists. While these clusters provide a variety of professional services, challenges persist due to vacant practices and regional disparities. To ensure sustainability, future efforts should strengthen digital health integration and promote the widespread adoption of innovative services provided by GP clusters.

## Introduction

Since 1990, primary care in Hungary has been generally provided by individual general practitioners (GP) and service providers, who operate under contracts with local municipalities (1–3). The local municipality is responsible for providing the infrastructure necessary for operating a GP practice, while GP services are financed by the National Health Insurance Fund (NHIF). In recent years, the financing method has been expanded to include performance and prevention incentives in addition to the per capita fee (4, 5). GPs play an important gatekeeper role in the healthcare system, treating patients with acute conditions, participating in the care of chronic patients, providing preventive services (such as vaccinations), issuing sick leave certificates, and, if necessary, referring patients to specialists (3, 6).

In developed countries, improving primary care has become a key health policy objective in recent decades, to enhance the population's access to health and preventive services close to their place of residence (7–11). In many countries, group practices and clusters have been established to promote various levels of professional and/or economic cooperation/integration among GPs, dentists, and other public health service providers operating in primary care (8, 12–17). These local and national model programs, as well as health policy reforms, also aim to coordinate and streamline healthcare services and patient pathways.

In the early 2010s, health policy experts in Hungary identified challenges similar to those in developed countries: the growing shortage of GPs hindered the population's access to primary care services, especially in areas where there were permanently vacant GP practices. Another challenge identified was that certain preventive and screening activities were less accessible in Hungarian GP practices (18).

From the mid-2010s, several pilot model programs were tested in Hungary to explore possible forms of operation for GP clusters, as well as their service and cooperation opportunities with primary care providers (18–20). Based on the Hungarian model programs and international experience, a nationwide system was established in June 2021 to provide higher-level healthcare services through the creation of GP clusters (21, 22). According to the law regulating this health policy measure, GP clusters can be established within the district as an administrative unit, with at least five GP practices joining together as a general rule (23). The development aims to improve the population's access to primary care services and to improve the quality of these services. The aim of establishing GP clusters is also to develop cooperation between the various actors involved in primary care (such as GPs, dentists, dental clusters, Health Promotion Offices (HPOs), Health Visitor Officers (HVOs), pharmacies, dieticians, physiotherapists, and other healthcare providers) (24).

**Figure 1:**
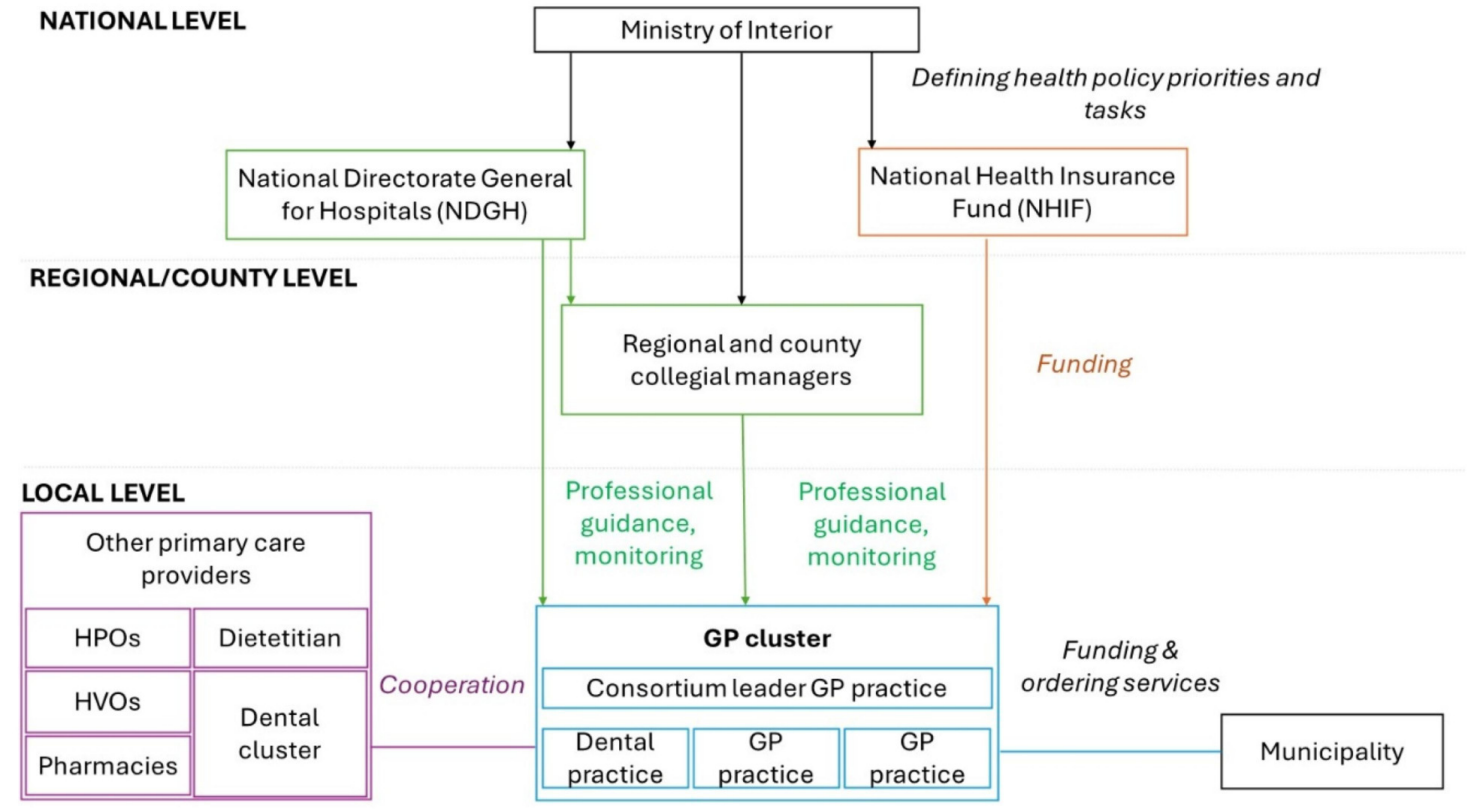
Simplified operational model of the GP clusters in Hungary. Source: Authors.

The National Directorate General for Hospitals (NDGH) is responsible for managing GP clusters at the national level, providing methodological guidelines for clusters (Figure 1). The NHIF provides additional funding for GP practices participating in GP clusters (5, 23).

County and regional collegial leaders perform coordination and professional support roles between national and local professional management (25).

There are three forms of cooperation available for establishing GP clusters: a) a united GP cluster; b) an integrated GP cluster; c) a consortium of GP practices (23). In a united GP cluster, the participating service providers merge into a jointly established healthcare provider, thereby ceasing their economic independence. In an integrated GP cluster, a service provider is also established jointly, but their economic independence does not cease; they coordinate their professional activities more actively. In a consortium of GP practices, service providers retain their professional and economic independence in their entirety. However, they establish a consortium cooperation agreement under the terms of which they coordinate their activities.

A defined number of preventive service hours must be provided every week in the GP clusters. Affiliated GPs can offer more than 70 types of professional services, such as vaccinations, screening for chronic diseases, mobilisation for organised screenings, minimal intervention, and lifestyle counselling (26).

Despite the nationwide introduction of GP clusters in Hungary in 2021, empirical evidence on their functioning remains scarce. The aim of the present study is to address this gap by providing a comprehensive description of GP practices and clusters between 2021 and 2024, and by analysing the determinants of cluster participation. From a theoretical perspective, the establishment and adoption of GP clusters can be interpreted through the lens of organizational and health policy diffusion theories (27). Our analysis was guided by the assumption that cluster membership may be influenced by multilevel contextual factors operating at practice, settlement, and district levels.

According to organizational theory, healthcare providers may respond to regulatory reforms and financial incentives in ways determined by institutional pressures, professional norms, and resource dependence. From this perspective, joining a GP cluster can be seen as an adaptive organisational response aimed at securing additional resources, professional development, and cooperation within a reorganized primary care system.

Based on the diffusion theory of health policy, the adoption of new organizational forms is influenced by contextual factors such as the density of service providers, spatial proximity, and administrative capacity. In addition, new forms of operation and novel procedures are more likely to spread where interaction between service providers is frequent and where the existence of a critical mass facilitates collective action.

Based on these theoretical perspectives, we formulated the following expectations:

1. Participation in GP clusters is more likely to occur in contexts where the density of service providers is higher, which means lower transaction costs when implementing cooperation.
2. Participation may also be shaped by identifiable territorial characteristics at the municipal and district levels.
3. Individual-level characteristics may also play a role in the early stages of policy implementation.

These theoretical expectations served as the basis for both the selection of variables and the multilevel modelling strategy used in the study.

## Methods

### Data collection

Based on the research permit issued by the National Directorate General for Hospitals (NDGH; No. OKFŐ/30565-4/2025), data on GP practices were requested in March 2025. The dataset included:

- Number of primary care GP practices (2019–2024), by year, county, and district.
- Number of vacant GP practices (2019–2024), by year, county, and district.
- Number of Hungarian and foreign citizens obtaining a medical degree in Hungary (2010–2024), by year and qualification.
- Number of graduates registered in the operational register (2010–2024), by qualification and year.
- Number of GPs registered in Hungary (2010–2024), by year.
- Number of GP practices (2021–2024), by county and district.

Additional NDGH data covered GP clusters and participating practices:

- Number of clusters and participating GPs (2021–2024), by cluster type, practice type (adult, paediatric, mixed), county, and district.
- Number of participating dental practices that have joined a GP cluster (2021-2024), by cluster type, practice type (adult, paediatric, mixed), county and district
- Number of professional services provided by clusters (2021–2024), by reimbursable activity, year, county, district, and cluster.
- Number of GPs operating in clusters in 2024, by county, district, and cluster.

### Data management

Following cleaning and validation of the NDGH database, we expanded it with regional information on municipalities, districts, counties, and regions, as well as population and development indicators. Additional practice-level data (status and type of practice) were obtained from publicly available databases of the National Health Insurance Fund (NHIF) and the Hungarian Central Statistical Office (HCSO) (retrieved 25 May 2025). The final database was managed and analysed using Stata 19.0 BE-Basic Edition.

## Methods for describing the functioning of GP practices and GP clusters

Information on GP training for the period 2010–2024, data on GP practices operating in primary care for the period 2019–2024, data on clusters for the period between 2021 and 2024, and the characteristics of the professional services provided by clusters in 2024 using statistical methods (frequency, average, median, ratio, and trends). We have provided a map showing the proportion of GPs affiliated with clusters by county.

## Methods for analysing factors associated with joining a GP cluster

### Development of variables

In line with our research question, the dependent variable in the analysis was membership of a GP cluster (binary variable: cluster member yes/no). In our model, we treated GP cluster participation as cross-sectional, cumulative binary outcome in the first 4 years after introduction of the law (cumulative participation between 2021 and 2024). The selection and structuring of independent variables were theoretically determined using organizational and health policy diffusion frameworks. The practice-level variables describe the characteristics of individual organizations. The settlement-level variables characterize the local environment and administrative context. District-level variables describe the broader structural opportunities that facilitate or constrain the creation of GP clusters. This multi-level specification is consistent with the theoretical assumption that the introduction of GP clusters and innovations in health systems is shaped not by isolated individual decision-making, but by interlocking contextual environments.

We developed the independent variables on three levels. At the level of GPs, the gender of the GP and the type of practice (adult, paediatric, mixed) were available. For the settlements designated as the operational headquarters of GP practices, the following data were available: the legal status of the settlements (village, small town, town, county town, county capital, capital district), the population of the settlement, the number of GPs per capita), and from the data available on the district (level of development of the district, population of the district, number of GPs per capita).

### Statistical analysis

Vacant GP practices were excluded from the regression analysis, as cluster participation is not meaningful in their case as in the absence of an operating GP, vacant practices cannot actively decide to join or not join a cluster. For comparison, vacant and active practices were analysed using two-sample *t*-tests for continuous variables and chi-square tests for categorical variables, with statistical significance defined as *p* < 0.05. In the descriptive analysis, practices that joined a cluster were compared with those that did not, applying the same tests and significance threshold. GP clusters operate within a territorial hierarchy: practices are located in settlements or groups of settlements, clusters are formed at the district level, and districts are nested within counties. To account for this embedded structure, we applied multilevel (hierarchical, mixed-effect) multivariate logistic regression models using Stata 19.0 BE-Basic Edition (28). Incorporating random effects ensured more accurate estimation of fixed effects and their standard errors. As a first step, empty random-intercept models were estimated at the settlement, district, and county levels to assess spatial clustering of cluster membership. A combined model was also tested, embedding settlements within districts. Intraclass correlation coefficients (ICC) with confidence intervals were calculated to quantify variance attributable to each territorial level (29). Empty and full models were compared using likelihood ratio tests, as well as changes in Akaike's Information Criterion (AIC) and Bayesian Information Criterion (BIC).

Multicollinearity among continuous variables was assessed using Pearson's correlation and variance inflation factors (VIF). Correlations above *r* = 0.5 were considered moderate, and above *r* = 0.7 strong; VIF values ≤5 indicated acceptable independence (30, 31). Model building proceeded stepwise: first practice-level variables, then settlement-level, and finally district-level variables were added. Expanded models were evaluated using likelihood ratio tests and changes in AIC and BIC. To explore potential non-linear relationships, continuous contextual variables (settlement population, district population, and GP density) were categorised into categorical variables (quartiles) to facilitate interpretation.

## Results

### The human resources (HR) state of GP practices and trends in GP training in Hungary

In Hungary, the number of GP practices operating in the framework of the social security system ranged between 6,251 and 6,347 in the period between 2019 and 2024 (Table 1). The proportion of vacant practices more than doubled from 7.31% in 2019 to 14.78% by the end of 2024. There is a steadily increasing and statistically significant trend in the proportion of vacant paediatric, mixed and all general practices (*p* for trend <0.001, respectively). In 2024, 9.71% of adult general practices, 15.22% of paediatric practices, and 25.84% of mixed practices were vacant.

**Table 1:** Number and proportion of GP practices and vacant practices 2019-2024, by practice type.

	2019	2020	2021	2022	2023	2024	*p* for trend
Number of general practices	6,347	6,251	6,347	6,340	6,340	6,339	0.545
Active practices by type	Number (proportion in %)						
Adult	3,247 (96.52)	3,214 (95.46)	3,189 (94.63)	3,172 (94.18)	3,110 (92.37)	3,041 (90.29)	0.003*
Paediatric	1,382 (92.63)	1,358 (91.08)	1,335 (89.72)	1,321 (89.02)	1,303 (87.74)	1,259 (84.78)	<0.001*
Mixed	1,254 (84.10)	1,208 (80.91)	1,176 (78.98)	1,167 (78.43)	1,124 (75.54)	1,102 (74.16)	<0.001*
Total	5,883 (92.69)	5,780 (91.01)	5,700 (89.81)	5,660 (89.27)	5,537 (86.92)	5,402 (85.22)	<0.001*
Vacant practices by type	Number (proportion in %)						
Adult	117 (3.48)	153 (4.54)	181 (5.37)	196 (5.82)	257 (7.63)	327 (9.71)	0.003*
Paediatric	110 (7.37)	133 (8.92)	153 (10.28)	163 (10.98)	182 (12.26)	226 (15.22)	<0.001*
Mixed	237 (15.90)	285 (19.09)	313 (21.02)	321 (21.57)	364 (24.46)	384 (25.84)	<0.001*
Total	464 (7.31)	571 (8.99)	647 (10.19)	680 (10.73)	803 (13.08)	937 (14.78)	<0.001*

* *p* < 0.05.

Sources: NDGH and the authors.

Considerable geographical disparities can be identified in Hungary regarding the proportion of vacant GP practices (Supplementary Table S1). In comparison, Budapest and Csongrád-Csanád County are in the best position, with the proportion of vacant practices increasing from 2% and 4% in 2019 to 8% and 7% in 2024, respectively. In contrast, in Tolna and Nógrád counties, which face substantial challenges, the proportion of vacant GP practices increased from 15% and 17% in 2019 to 26% and 29% in 2024.

Compared to 2010, the number of Hungarian and foreign doctors (26,365) registered in the NDGH's healthcare HR database who graduated in Hungary increased by nearly 20% (32,530) by 2024 (Supplementary Table S2). The proportion of foreign doctors increased from 0.4% in 2010 to 3.4% in 2024. The number of Hungarian and foreign doctors with professional qualifications listed in the database also increased steadily between 2010 and 2024 (except for those in general practice). The number of Hungarian and foreign doctors with general practitioner qualifications ranged from 4,216 to 4,839, and the proportion of foreign doctors with this qualification remained stable during the period under review. The proportion of foreign doctors with a qualification in general practice remained stagnant at 0.2% during the period under review, indicating that foreign doctors registered in Hungary in increasing numbers preferred other specialities. The number of persons with valid general practitioner registration fluctuated between 4,717 and 5,515, with no clear upward or downward trend evident between 2010 and 2024.

Type, number, and HR status of GP clusters between 2021 and 2024.

The number of GP clusters established in Hungary increased from 365 in 2021 to 422 in 2024 (Table 2). During this period, one GP cluster operated in a unified form in Szigetszentmiklós district, while all other clusters operated in a consortium form. No integrated cluster has been established in the three years since the introduction of the cluster model in 2021.

**Table 2:** Number and proportion (%) of GP clusters by operating form, and number and proportion of joined GP practices by practice type between 2021 and 2024.

	2021	2022	2023	2024	*p* for trend
Number of GP clusters	365	388	411	422	0.011*
Number and proportion (%) of GP clusters operating as consortium (%)	364 (99.73)	387 (99.74)	410 (99.76)	421 (99.76)	0.053
Number and proportion (%) of GP clusters operating as a unified entity (%)	1 (0.27)	1 (0.26)	1 (0.24)	1 (0.24)	0.053
Number and proportion (%) of GP clusters established exclusively with GPs, compared to all GP clusters	248 (67.95)	264 (68.04)	285 (69.34)	293 (69.43)	0.078
Number and proportion (%) of GP clusters established with GPs and dental practices, compared to all GP clusters	117 (32.05)	124 (31.96)	126 (30.66)	129 (30.57)	0.078
Number of GP practices that have joined a cluster	2,417	2,790	2,884	3,147	0.024*
Number and proportion (%) of GP practices that have joined a cluster, compared to all joined practices	2,296 (94.99)	2,656 (95.20)	2,749 (95.32)	3,010 (95.65)	0.017*
Number and proportion (%) of dental practices that have joined a cluster, compared to all joined practices	121 (5.01)	134 (4.80)	135 (4.68)	137 (4.35)	0.017*
Number and proportion (%) of adult practices that have joined a cluster compared to all joined practices	1,337 (55.32)	1,519 (54.44)	1,658 (57.49)	1,760 (55.93)	0.509
Number and proportion (%) of paediatric practices that have joined a cluster compared to all joined practices	618 (25.57)	655 (23.48)	684 (23.72)	716 (22.75)	0.115
Number and proportion (%) of mixed practices that have joined a cluster compared to all joined practices	462 (19.11)	616 (22.08)	542 (18.79)	671 (21.32)	0.734
Average number of practices in a GP cluster	6.62	6.93	7.27	7.65	0.001*
Median number of practices in a GP cluster	6	7	7	7	0.225

* *p* < 0.05.

Sources: NDGH and the authors.

Between 2021 and 2024, approximately one-third of GP clusters typically involved one or two dentists. By 2024, a total of 137 dental practices had joined GP clusters, accounting for 4% of all affiliated practices.

In 2021, 55% of the GP practices that joined GP clusters were adult practices, 26% were paediatric practices, and 19% were mixed practices. By 2024, the proportion of practice types within all GP clusters had not changed significantly. While in 2021 there were an average of 6.62 and a median of 6 practices in each GP cluster, by 2024 there were an average of 7.65 and a median of 7 practices working together in GP clusters. While in 2021, 38% of all GP practices joined clusters, by 2024, nearly half (47%) of practices had joined one of the 422 GP clusters.

**Figure 2:**
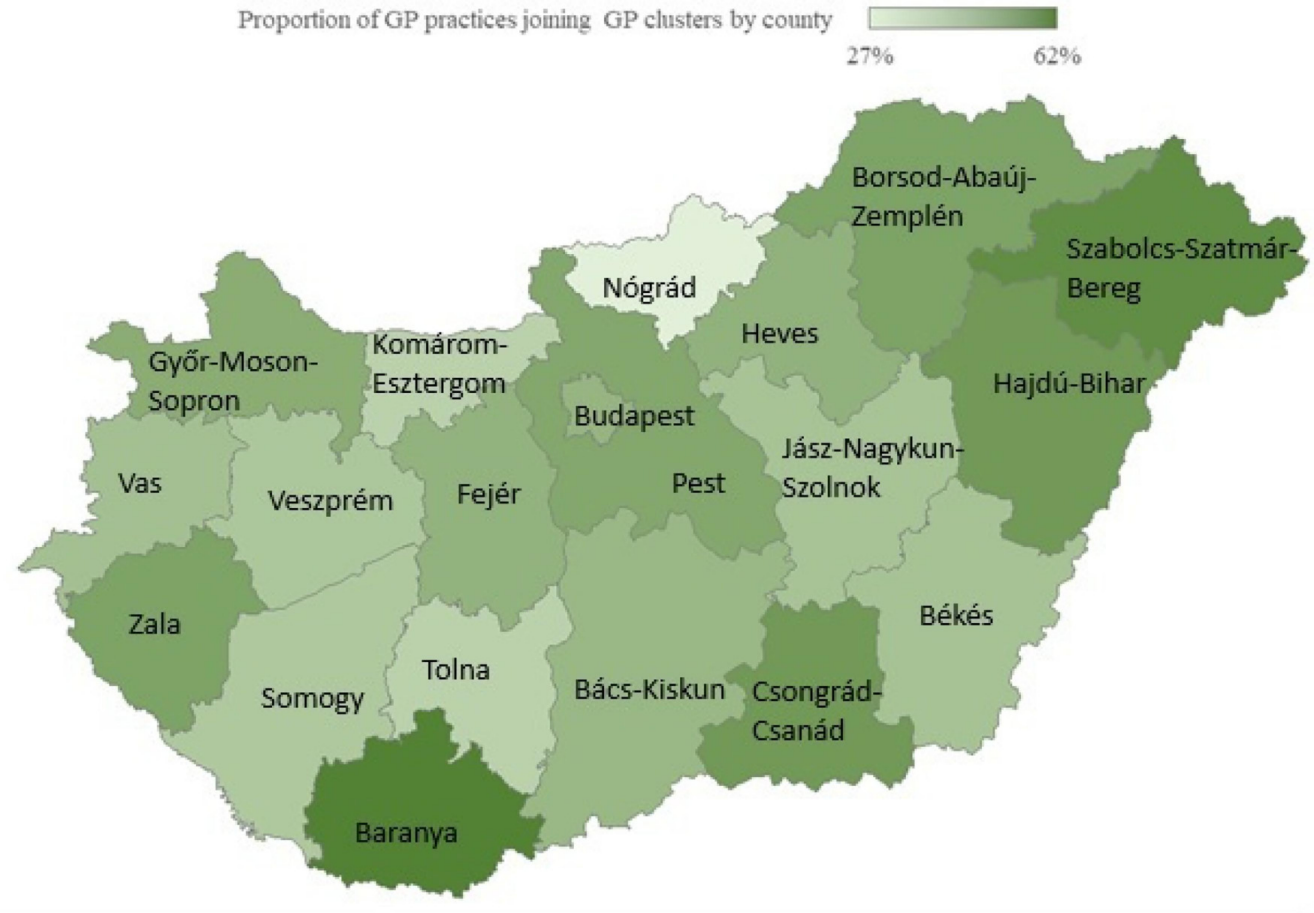
The proportion of GP practices joining GP clusters by county in 2024. Sources: NDGH and the authors.

In terms of the proportion of GP practices that joined GP clusters, substantial regional disparities were identified in 2024 (Figure 2). The lowest proportion of GPs joining clusters was observed in Nógrád (27%), Esztergom (36%), and Tolna (37%) counties, while the highest proportion was found in Baranya (62%), Szabolcs-Szatmár-Bereg, and Csongrád-Csanád (55%).

In the capital, 46% of GPs joined a GP cluster, which was in line with the national average (47%) (Supplementary Table S3). Compared to 2021, the proportion of practices joining GP clusters increased steadily in almost all counties, with the sole exception of Zala County, where a minimal decrease of 1% was observed compared to 2022.

In 2024, 17% of the 3,147 GP practices that joined the 422 GP clusters operated in the capital, and 12% in Pest County (Supplementary Table S4).

#### Professional activities provided by GP clusters

In 2024, GP clusters reported providing 78 different professional activities to NDGH, with a total of 1,964,436 preventive services provided during the year (Supplementary Table S5). The five most frequently reported professional activities (1. Pulse oximetry test, 2. Vaccination at a GP's office, 3. Body composition assessment, 4. 12-lead electrocardiogram (ECG) and evaluation for clinically relevant reasons, and 5. Screening for obstructive sleep apnoea syndrome using questionnaires, such as STOP BANG and Berlin questionnaires, Epworth Sleepiness Scale) accounted for 66.2% of all reported services. Various Point-of-care tests (POCT) accounted for 10.3% of all preventive services, while various telemedicine services accounted for 6.5% of all reported services.

#### Analysis of factors associated with a GP's participation in a cluster

A total of 6,421 GP practices were included in the database, representing all GP practices contracted to the NHIF in the country in May 2025. Of these GP practices, 956 were vacant, and these were omitted from the analysis database after comparison. In the case of vacant practices, cluster participation is not a meaningful choice in the absence of an operating GP as vacant practices cannot actively decide to join or not join a cluster. A comparison of active and vacant GP practices is provided in Supplementary Table S6. Compared to active practices, vacant practices had a higher proportion of mixed practices, less developed districts and villages, and the population of the settlements and districts in vacant practices was significantly smaller than that of the active practices.

A total of 5,465 GP practices, 197 districts, and 1,451 municipalities participated in the analysis. Of the 5,465 practices, 3,002 joined GP clusters. The Supplementary Table S7 shows the variables according to the status of joining a GP cluster. During the collinearity test of continuous independent variables, the population of the settlement and the population of the district showed a strong correlation (*r* = 0.74, *p*-value <0.001). The variance inflation factor was less than 5 for all continuous independent variables.

Table 3 summarises the results of empty models containing only random intercepts for the following indicators: variance estimates, intraclass correlation coefficients (ICCs), model fit statistics [Akaike Information Criterion (AIC) and Bayesian Information Criterion (BIC)], and likelihood ratio tests (LR test) compared to simpler models.

**Table 3:** Variance components, intraclass correlation coefficients (ICC), and fit statistics from empty random-intercept models at county, district, and settlement levels.

Level	Variance	ICC	AIC	BIC	LR test *p*-value
County	0.072	0.021	7,471.524	7,484.737	<0.001*
District	1.105	0.251	7,041.848	7,055.060	<0.001*
Settlement	2.924	0.471	6,912.168	6,925.380	<0.001*
Settlement nested in district	2.129	0.514	6,824.742	6,844.560	<0.001*

* *p* < 0.05.

The largest variance component and ICC were observed at the settlement level; in the empty model, approximately 47% of the residual variance in joining a GP cluster can be attributed to differences between settlements. The district level also showed significant clustering (ICC = 0.25), while the degree of clustering at the county level was negligible (ICC = 0.02). Taking into account the embedded structure of settlements within districts in the combined model significantly improved the model fit (lowest AIC and BIC). In the combined model, the variance at the district level was 1.35 (SE = 0.28), with an ICC of 0.20 (95% CI: 0.14–0.27). Meanwhile, the variance within settlements within the district was 2.13 (SE = 0.33), corresponding to an ICC of 0.51 (95% CI: 0.45–0.58). The higher variance and ICC at the settlement level indicate that approximately 51% of the residual variance in joining the GP cluster is attributed to differences between settlements within districts. Likelihood ratio tests confirmed that multilevel random intercept models, including those that considered settlement and district separately and together, significantly improved the fit compared to the simpler model that did not account for cluster effects (*p*-value <0.001).

When practice, settlement, and district-level variables were added to the empty model, the results showed the following. The model containing the two available practice-level variables (GP's gender, practice type) did not show significantly better model fit compared to the empty model. Gender (male vs. female: OR = 1.02, *p* = 0.786) and practice type (paediatric practice: OR = 1.02, *p* = 0.833; mixed practice: OR = 0.92, *p* = 0.525 vs. adult practice) did not show a significant association with joining GP clusters. Adding the available two practice-level variables did not significantly improve the fit of the model (LR test *p* = 0.912), and beyond contextual effects, the impact of individual practices proved to be minimal. When adding settlement- and district-level variables to the intermediate model containing practice-level variables, the number of GPs per capita showed a correlation with joining a GP cluster (OR = 0.67, *p* < 0.01). Among the district indicators, the number of GPs per capita was associated with joining a GP cluster (*p* < 0.01), indicating that districts with a higher density of GPs are more likely to join clusters. The addition of district-level variables significantly improves the fit of the model compared to the model containing only settlement variables (LR test *p* < 0.001).

In developing the final model, we excluded practice-level variables and converted continuous variables into categorical variables (quartiles) to examine nonlinear effects.

In the final model (Table 4), the quartile classification of GP density showed that practices in the second quartile had a higher chance of joining a GP cluster compared to those in the lowest quartile (OR = 1.49; 95% CI, 0.99–2.23; *p* = 0.056), although no significant association was found for the higher quartiles. The population and type of settlement also did not show a significant effect, for example in the districts of the capital, where the probability of joining a cluster was lower (OR = 0.30; 95% CI, 0.09–1.02; *p* = 0.055). District-level variables showed the following correlations: in districts in the third quartile for GP density, the probability of cluster membership was twice as high (OR = 2.15; 95% CI, 1.23–3.75; *p* = 0.007), which increased further in the highest quartile (OR = 2.82; 95% CI, 1.39–5.74; *p* = 0.004). The district population showed a positive but marginally significant association in the highest quartile (OR = 2.22; 95% CI, 0.94–5.27; *p* = 0.069). The level of development of the district was not significantly associated with the concentration of GP clusters in our model. The random intersection variance components showed significant residual heterogeneity at both the district (variance = 1.00; ICC = 0.16) and settlement levels (variance = 2.03; ICC = 0.48). This demonstrates the importance of considering hierarchical clustering and suggests that unmeasured contextual effects may be at play. The final model test showed a good fit: Wald *χ*^2^(20) = 36.52, *p* = 0.0134.

**Table 4:** Results of the final multilevel logistic regression model on GP cluster participation.

Variable	Category	Odds ratio (OR)	Std. error	*Z*	*p*-value	95% confidence interval
Settlement GP density (quartiles)	2	1.49	0.31	1.91	0.056	0.99–2.23
	3	1.27	0.29	1.04	0.296	0.81–1.98
	4	0.91	0.18	−0.46	0.643	0.63–1.33
Settlement population (quartiles)	2	1.26	0.31	0.94	0.348	0.78–2.03
	3	1.06	0.41	0.16	0.873	0.50–2.27
	4	1.09	0.72	0.13	0.893	0.30–3.99
District population (quartiles)	2	1.25	0.31	0.89	0.371	0.77–2.04
	3	1.64	0.55	1.48	0.138	0.85–3.17
	4	2.22	0.98	1.82	0.069	0.94–5.27
District GP density (quartiles)	2	1.27	0.33	0.91	0.360	0.76–2.13
	3	2.15	0.61	2.68	0.007*	1.23–3.75
	4	2.82	1.02	2.87	0.004*	1.39–5.74
Settlement type	Small town	0.86	0.23	−0.57	0.570	0.51–1.45
	Town	1.06	0.26	0.23	0.817	0.65–1.72
	County town	0.93	0.68	−0.09	0.925	0.22–3.92
	County capital	0.78	0.47	−0.41	0.680	0.24–2.52
	Capital district	0.30	0.19	−1.92	0.055	0.09–1.02
District development level	Deprived	1.13	0.47	0.28	0.777	0.49–2.56
	Lesser developed	1.09	0.35	0.28	0.783	0.58–2.04
	Developed	1.16	0.39	0.43	0.665	0.60–2.22
Intercept		0.47	0.15	−2.40	0.016*	0.25–0.87
Random effects		Estimate		Std. error		95% confidence interval
District level		1.00		0.23		0.64–1.58
Settlement within district		2.03		0.32		1.49–2.76
Intraclass correlation coefficients (ICC)		Estimate		Std. Error		95% confidence interval
District level		0.16		0.03		0.11–0.23
Settlement within district		0.48		0.04		0.41–0.55

* *p* < 0.05.

## Discussion

The results of our study are consistent with those of previous research, which have found a shortage of GPs in primary care in Hungary. This, combined with the ageing of the population, poses further challenges and risks to the system's functioning (32, 33). The continuous, trend-like increase in the proportion of vacant GP practices also indicates the systemic risk.

Our modelling analysis aimed at modelling GP cluster participation as cross-sectional outcome by taking into account the hierarchical nature of the data. We focussed on the early years of implementation thus it is cross-sectional in nature with respect to cumulative participation by 2024. As such, it does not capture the temporal dynamics of cluster adoption, including early vs. late adopters, diffusion processes, or time-varying policy incentives which may become more important with time in the future. Later on or with relevant further incentives introduced during implementation, longitudinal approaches, such as event-history models or panel regression would better suit to analysing these dynamics but were beyond the scope of the present study.

Interpreting the results based on our theoretical framework, they partially support diffusion-based expectations. The significant role of district-level GP density is consistent with the assumption that adaptation is facilitated by a critical mass of service providers and opportunities for interaction within professional networks. This supports the theoretical assumption that organizational innovations spread more easily under structurally favourable conditions.

The results highlight the role of territorial and service-provider density factors in joining a GP cluster, with settlement- and district-level contexts emerging as determinants. A higher density of GPs at the district level significantly increased the likelihood of joining GP clusters. The observed associations involving GP density and population however should be interpreted with caution, and as a descriptive pattern rather than a causal policy effect due to the limitations of the present analysis. The finding that GP clusters were more common in districts with higher GP density may indicate that better-resourced areas were more likely to adopt the cluster model.

Despite taking available contextual factors into account, the significant residual clustering observed at both the municipal and district levels suggests that unmeasured local conditions and organizational characteristics may further shape the behaviour of joining GP clusters. High settlement-level ICC further supports the theoretical assumption that the local institutional environment can strongly influence organizational behaviour. From an organizational perspective, the formation of such clusters may reflect local norms, informal cooperation mechanisms, and a shared interpretation of policy incentives. Our results support the interpretation that participation in clusters may not be solely an individual strategic decision, but rather a phenomenon embedded in the territorial institutional context.

Notably, our findings reflect the limited set of physician and practice-level variables available in our dataset (doctor's gender and practice type). At the same time, the exceptionally high ICCs at the settlement level indicate that unmeasured practice-level or local organisational characteristics are extremely important. For example, studies show that practice-level characteristics (such as age and sex of physicians) and/or the composition of patients in the practice (by income, rural/urban, etc.) are important characteristics associated with joining team-based primary care model in Canada (34, 35).

The absence of a statistically significant association between district development level and cluster participation indicates that socioeconomic disadvantage, as measured by the development index used in this study, does not independently predict adoption within the multilevel framework. Therefore, interpretations suggesting direct socioeconomic inequality effects should be made cautiously and remain strictly aligned with the statistical evidence presented. The results underscore the importance of considering multiple contextual factors in understanding the consolidation of GP clusters when designing health policy measures to support the effective optimisation of access to care.

Following the direction of primary care improvements in developed countries, GP clusters have also appeared at the national level in Hungary since 2021. While in some countries, such as the United Kingdom, Canada and Australia, primary care clusters have developed extensive cooperation between multiple service providers (GPs, dentists, specialist care, pharmacies, other public health actors), in other countries, such as Germany, primary care clusters have been formed exclusively between GPs or exclusively between dentists (8, 11, 13, 14, 36). A unique practice has developed in Hungary, where only dentists have joined the dental clusters established in 2021. In contrast, our research reveals that in one-third of GP clusters, both GPs and dentists have joined the clusters and are cooperating (22).

In comparison to the Hungarian model, there has been more extensive and active cooperation in primary care clusters in the United Kingdom and Italy (14, 16). Numerous studies have confirmed that cooperation between GPs and dentists is beneficial in terms of prevention and patient care, yielding measurable societal benefits (12, 36). Cooperation between primary care providers can create opportunities to improve the quality of healthcare services and develop coordinated patient pathways (37). However, operating primary care clusters poses challenges in terms of innovative financing techniques and securing adequate human resources and equipment. The results of our research, in line with a Hungarian study conducted in 2024, also highlighted that modern digital health technologies (telemedicine, remote monitoring) and diagnostics using modern POCT devices have also appeared in the GP clusters. However, these are still present at a low rate compared to all services (1).

Our study reached a similar conclusion to another analysis examining the operation of Hungarian dental clusters, which found that dentists working in primary care were only willing to choose a consortium cluster format that would allow them to retain their economic independence (22). According to our findings, Hungarian GPs also prefer the consortium format, which offers a greater degree of economic independence when establishing GP clusters (21).

Our study reached a similar conclusion to a study evaluating the operation of Hungarian dental clusters, which found that the size of the municipality and the density of service providers in a given administrative area significantly increased the likelihood of joining a cluster (22). According to the results of our current study, districts with a higher density of GPs are more likely to have GP clusters. Our findings are also significant from a health policy perspective, as they highlight the risk that the social benefits of GP clusters may be concentrated in administrative areas where the population already has better access to primary care services due to a higher density of GPs. Additional research is required also to establish effective strategies for encouraging GPs to participate in clusters. In addition to financial incentives, possible motivational tools include developing methodologies to support professional cooperation, creating innovative patient pathway systems, training human resources to provide additional services, and providing material resources (digital technologies, POCT devices) and improving infrastructure conditions.

### Limitations and future research areas

Our research has several important limitations to consider. The NDGH only had data available on GP training for the period from 2010 to 2024. We also only had access to data on the professional activities of GP clusters for the period 2021–2024, so we could only examine the first years of implementation. We were unable to compare GP practices with those prior to the establishment of GP clusters. There was also a lack of data on the status of equipment and infrastructure, development plans, and the number of partners and topics of collaborations.

Vacant practices account for a substantial share of the primary care system in Hungary and are more common in rural and socioeconomically disadvantaged areas. Excluding these practices may introduce selection bias and distort associations with district development and GP density. Accordingly, our results apply only to active practices and cannot be generalized to structurally disadvantaged areas.

Our multivariate regression analysis has several limitations that should be considered when interpreting the results. Cross-sectional design limits the drawing of causal conclusions, and residual confounding factors caused by variables that are not measured or available at multiple levels may distort the results despite taking hierarchical organization into account. The high settlement-level ICC indicates that unmeasured local factors may be important determinants of cluster participation. Examples include the age of the GP, practice-level characteristics that associated with GP cluster membership, such as organizational culture, characteristics of the GP district, and financial incentives, which were not available but may be important factors. Aggregate measures used as proxies for healthcare, such as the density of GP providers, may oversimplify the complex local environment and motivations. These proxies cannot capture qualitative differences such as service quality or patient preferences, which limits generalizability.

Similarly, the strong association observed for district-level GP density suggests that a critical mass of providers may facilitate cluster formation. Higher GP density may lower transaction costs of collaboration, increase peer influence, and create competitive or normative pressures to join. However, in low-density districts, structural constraints may limit the feasibility of forming clusters. These findings imply that cluster policy may operate differently depending on local service ecology, and that uniform national incentives may produce territorially differentiated outcomes.

Given the very limited set of practice-level variables available (gender and practice type), our analysis does not allow robust conclusions regarding individual-level determinants of cluster participation. The absence of statistically significant practice-level effects should therefore not be interpreted as evidence that individual motivations or professional characteristics are irrelevant. Future research incorporating richer practice-level information, such as staffing, workload, organisational structure, or prior collaboration experience, practice size and composition may help explain the substantial unexplained variance observed at the settlement level.

Importantly, the cumulative cross-sectional design implies that our findings reflect statistical associations rather than causal effects. Because cluster participation was measured as a cumulative outcome by 2024, we cannot determine whether contextual characteristics preceded or resulted from cluster formation. Reverse causality and policy-driven temporal effects therefore cannot be excluded.

Another limitation is the exclusion of vacant practices from the regression analysis. Given that vacancies are disproportionately concentrated in socioeconomically disadvantaged and rural areas, the associations observed, particularly those related to district development and GP density, may underestimate structural barriers in these regions. The results should therefore be generalised with caution to areas with high vacancy rates. Future analyses incorporating vacant practices, e.g., modelling vacancy as an outcome or including vacancy rates as contextual predictors, may provide a more complete picture of structural disadvantage.

Continuous contextual variables (e.g., settlement/district GP density and population size) were categorised into quartiles, which may have introduced arbitrary cut-points and reduced information. In addition, effect estimates may be sensitive to the underlying data distribution and the chosen cut-points, which can limit comparability and robustness. Although this approach was chosen to explore potential non-linearities, it limits precision and should be refined in future analyses using spline-based or polynomial models.

Our regression analysis treats cluster participation as a cumulative outcome during the early years of implementation and does not incorporate the timing of adoption. This limits our ability to examine longitudinal processes such as diffusion, early adoption, or policy-driven temporal effects at present, and is beyond the scope of the present analysis. In the future, with more years of implementation or with extra incentives introduced during the process, longitudinal event-history or panel models would be better suited to analysing adoption dynamics but were beyond the scope of the present study.

A future line of research in this area could be to analyse cooperation between GPs, dentists, and Health Promotion Offices (38). Another area of research could be the impact of the COVID-19 pandemic on the functioning of the domestic healthcare system and the potential for expanding digital health technologies in GP practices (39–42).

## Conclusions

In this study, we examined the development of GP clusters in Hungary, a strategic direction aimed at strengthening primary healthcare. The proportion of vacant GP practices has risen steadily, reaching 15% by 2024, while substantial regional disparities have also emerged. Between 2021 and 2024, the number of GP clusters has grown steadily at the national level. By 2024, nearly half of all GPs had joined a cluster. On average, there were seven GP practices in each cluster, and in one-third of the clusters, GP and dentist collaborations had also been established. More than 70 types of professional activities are available in the GP clusters portfolio, but the proportion of modern digital health technologies and POCT is still low. While GP clusters have expanded nationwide, participation appears higher in districts with greater GP density. Whether this reflects broader socioeconomic differences requires further investigation. The results of the period examined in the analysis highlight that health policy measures aimed at developing primary care should focus on the widespread adoption of innovative services provided by GP clusters and solving the problem of unfilled practices.

## Data Availability

The datasets presented in this article are not readily available because the study and the data provision have been authorised by the National Directorate General for Hospitals on the basis of the research application “OKFŐ/30565-4/2025”, approved on 5 March 2025. All relevant data may be obtained from the corresponding author upon a reasonable request. Requests to access the datasets should be directed to Gergő Túri, turi.gergo@semmelweis.hu.
