# Supplementary material for: The development of the primary care general practitioner cluster model: experiences from Hungary
Source: Front Health Serv. 2026 Mar 30;6:1769211. doi: 10.3389/frhs.2026.1769211 (PMC13070958; doi:10.3389/frhs.2026.1769211)
Supplement: Supplementary file 1 [file Table1.docx]

The development of the primary care general practitioner cluster model: experiences from Hungary

**Supplementary Table 1.** Number and percentage of vacant GP practices by county, 2019–2024.

| County | 2019 | 2020 | 2021 | 2022 | 2023 | 2024 |
| --- | --- | --- | --- | --- | --- | --- |
| Bács-Kiskun | 32 (9%) | 36 (11%) | 31 (9%) | 31 (9%) | 39 (11%) | 47 (14%) |
| Baranya | 10 (4%) | 14 (5%) | 18 (7%) | 21 (8%) | 31 (11%) | 27 (10%) |
| Békés | 32 (13%) | 35 (14%) | 40 (16%) | 45 (19%) | 51 (21%) | 61 (25%) |
| Borsod-Abaúj-Zemplén | 59 (13%) | 67 (14%) | 75 (16%) | 76 (16%) | 90 (19%) | 102 (22%) |
| Budapest | 25 (2%) | 28 (2%) | 46 (4%) | 57 (5%) | 63 (5%) | 94 (8%) |
| Csongrád-Csanád | 12 (4%) | 19 (7%) | 21 (8%) | 17 (6%) | 18 (7%) | 18 (7%) |
| Fejér | 27 (11%) | 36 (14%) | 39 (15%) | 37 (15%) | 44 (17%) | 54 (21%) |
| Győr-Moson-Sopron | 16 (6%) | 17 (6%) | 15 (6%) | 14 (5%) | 27 (10%) | 34 (13%) |
| Hajdú-Bihar | 17 (5%) | 24 (7%) | 21 (6%) | 21 (6%) | 24 (7%) | 30 (9%) |
| Heves | 23 (12%) | 30 (15%) | 34 (17%) | 36 (18%) | 39 (20%) | 42 (21%) |
| Jász-Nagykun-Szolnok | 37 (15%) | 47 (19%) | 45 (18%) | 44 (18%) | 54 (22%) | 61 (24%) |
| Komárom-Esztergom | 8 (4%) | 14 (7%) | 17 (9%) | 21 (11%) | 26 (14%) | 31 (16%) |
| Nógrád | 23 (17%) | 23 (17%) | 33 (25%) | 34 (26%) | 33 (25%) | 38 (29%) |
| Pest | 39 (6%) | 45 (6%) | 58 (8%) | 57 (8%) | 72 (10%) | 81 (11%) |
| Somogy | 20 (9%) | 26 (12%) | 31 (15%) | 33 (15%) | 38 (18%) | 45 (21%) |
| Szabolcs-Szatmár-Bereg | 19 (6%) | 20 (6%) | 31 (9%) | 32 (10%) | 31 (9%) | 32 (10%) |
| Tolna | 24 (15%) | 29 (19%) | 25 (16%) | 27 (17%) | 37 (24%) | 41 (26%) |
| Vas | 17 (10%) | 21 (12%) | 23 (13%) | 32 (19%) | 30 (18%) | 35 (20%) |
| Veszprém | 13 (6%) | 20 (9%) | 24 (11%) | 26 (12%) | 34 (16%) | 38 (18%) |
| Zala | 11 (6%) | 20 (11%) | 20 (11%) | 19 (11%) | 22 (13%) | 26 (15%) |
| Total | **464 (7%)** | **571 (9%)** | **647 (10%)** | **680 (11%)** | **803 (13%)** | **937 (15%)** |

**Sources:** NDGH and the authors

**Supplementary Table 2.** Number and proportion of medical doctors listed in the health-care HR database between 2010-2023, by nationality and qualifications

|  | **Number and proportion (%) of medical doctors listed in the health-care HR database** | | | **Number and proportion of medical doctors with professional qualifications listed in the health-care HR database (except: general practitioners)*** | | | **Number and proportion of medical doctors with qualifications in general practice** | | | **Number of medical doctors with valid general practitioner registration on 31 December 2024** |
| --- | --- | --- | --- | --- | --- | --- | --- | --- | --- | --- |
| Year | Hungarian | Foreign nationals | All | Hungarian | Foreign nationals | All | Hungarian | Foreign nationals | All | All |
| **2010** | 26267 (99,6%) | 98 (0,4%) | 26 365 | 45789 (99,6%) | 191 (0,4%) | 45 980 | 4207 (99,8%) | 9 (0,2%) | 4 216 | 4 717 |
| **2011** | 26891 (99,6%) | 109 (0,4%) | 27 000 | 46344 (99,6%) | 195 (0,4%) | 46 539 | 4369 (99,8%) | 9 (0,2%) | 4 378 | 4 897 |
| **2012** | 28044 (99,4%) | 169 (0,6%) | 28 213 | 46736 (99,6%) | 198 (0,4%) | 46 934 | 4520 (99,8%) | 10 (0,2%) | 4 530 | 5 074 |
| **2013** | 28748 (99,3%) | 208 (0,7%) | 28 956 | 47068 (99,6%) | 205 (0,4%) | 47 273 | 4714 (99,8%) | 10 (0,2%) | 4 724 | 5 288 |
| **2014** | 29893 (98,9%) | 309 (1,1%) | 30 202 | 48369 (99,6%) | 208 (0,4%) | 48 577 | 4915 (99,9%) | 11 (0,2%) | 4 926 | 5 515 |
| **2015** | 27510 (98,6%) | 390 (1,4%) | 27 900 | 49244 (99,6%) | 217 (0,4%) | 49 461 | 4425 (99,9%) | 5 (0,1%) | 4 430 | 4 916 |
| **2016** | 28379 (98,3%) | 500 (1,7%) | 28 879 | 49444 (99,6%) | 221 (0,4%) | 49 665 | 4624 (99,8%) | 7 (0,1%) | 4 631 | 5 143 |
| **2017** | 29122 (97,9%) | 624 (2,1%) | 29 746 | 50971 (99,6%) | 227 (0,4%) | 51 198 | 4773 (99,9%) | 7 (0,1%) | 4 780 | 5 312 |
| **2018** | 29669 (97,5%) | 746 (2,5%) | 30 415 | 51328 (99,6%) | 229 (0,4%) | 51 557 | 4860 (99,8%) | 10 (0,2%) | 4 870 | 5 408 |
| **2019** | 30418 (97,4%) | 828 (2,6%) | 31 246 | 51761 (99,5%) | 239 (0,5%) | 52 000 | 4987 (99,8%) | 10 (0,2%) | 4 997 | 5 545 |
| **2020** | 27292 (96,7%) | 917 (3,3%) | 28 209 | 53096 (99,5%) | 250 (0,5%) | 53 346 | 4133 (99,8%) | 8 (0,2%) | 4 141 | 4 587 |
| **2021** | 28477 (96,6%) | 1015 (3,4%) | 29 492 | 53174 (99,5%) | 262 (0,5%) | 53 436 | 4318 (99,8%) | 8 (0,2%) | 4 326 | 4 783 |
| **2022** | 29827 (96,9%) | 935 (3,1%) | 30 762 | 53261 (99,5%) | 269 (0,5%) | 53 530 | 4605 (99,8%) | 9 (0,2%) | 4 614 | 5 092 |
| **2023** | 30872 (96,8%) | 1021 (3,2%) | 31 893 | 53529 (99,5%) | 279 (0,5%) | 53 808 | 4774 (99,8%) | 8 (0,2%) | 4 782 | 5 284 |
| **2024** | 31437 (96,6%) | 1093 (3,4%) | 32 530 | 53951 (99,5%) | 289 (0,5%) | 54 240 | 4829 (99,8%) | 10 (0,2%) | 4 839 | 5 344 |

**Sources:** NDGH and the authors. *If a medical doctor has multiple professional qualifications, they will appear multiple times in the table.

**Supplementary Table 3.** Number and proportion (%) of general practitioners who have joined a GP cluster compared to all GP practices operating in the relevant county in 2021-2024.

| County | 2021 | 2022 | 2023 | 2024 |
| --- | --- | --- | --- | --- |
| Bács-Kiskun | 141 (41%) | 146 (43%) | 153 (45%) | 153 (45%) |
| Baranya | 125 (46%) | 128 (47%) | 138 (51%) | 169 (62%) |
| Békés | 82 (34%) | 84 (35%) | 87 (36%) | 100 (41%) |
| Borsod-Abaúj-Zemplén | 225 (49%) | 228 (49%) | 232 (50%) | 239 (52%) |
| Budapest | 407 (35%) | 459 (40%) | 475 (41%) | 531 (46%) |
| Csongrád-Csanád | 128 (47%) | 134 (50%) | 141 (53%) | 147 (55%) |
| Fejér | 96 (38%) | 104 (41%) | 107 (42%) | 118 (46%) |
| Győr-Moson-Sopron | 56 (21%) | 96 (36%) | 101 (38%) | 128 (48%) |
| Hajdú-Bihar | 156 (47%) | 172 (51%) | 173 (52%) | 183 (55%) |
| Heves | 84 (42%) | 91 (46%) | 92 (47%) | 92 (47%) |
| Jász-Nagykun-Szolnok | 70 (28%) | 91 (36%) | 93 (37%) | 103 (41%) |
| Komárom-Esztergom | 44 (23%) | 50 (26%) | 55 (29%) | 70 (36%) |
| Nógrád | 19 (14%) | 24 (18%) | 30 (23%) | 35 (27%) |
| Pest | 298 (42%) | 322 (45%) | 332 (46%) | 359 (50%) |
| Somogy | 61 (29%) | 75 (35%) | 79 (37%) | 84 (39%) |
| Szabolcs-Szatmár-Bereg | 178 (54%) | 177 (53%) | 179 (54%) | 195 (59%) |
| Tolna | 33 (21%) | 50 (32%) | 52 (33%) | 57 (37%) |
| Vas | 48 (28%) | 49 (29%) | 53 (31%) | 72 (42%) |
| Veszprém | 81 (38%) | 83 (39%) | 84 (40%) | 84 (40%) |
| Zala | 85 (48%) | 93 (53%) | 93 (53%) | 91 (52%) |
| All | 2417 (38%) | 2656 (42%) | 2749 (43%) | 3010 (47%) |

**Sources:** NDGH and the authors.

**Supplementary Table 4.** Number of general practitioners and dentists who joined GP clusters, and the proportion (%) of practices that joined per county compared to the total number of practices that joined in 2024.

| County | 2024 |
| --- | --- |
| Bács-Kiskun | 161 (5%) |
| Baranya | 181 (6%) |
| Békés | 105 (3%) |
| Borsod-Abaúj-Zemplén | 253 (8%) |
| Budapest | 543 (17%) |
| Csongrád-Csanád | 156 (5%) |
| Fejér | 122 (4%) |
| Győr-Moson-Sopron | 129 (4%) |
| Hajdú-Bihar | 195 (6%) |
| Heves | 98 (3%) |
| Jász-Nagykun-Szolnok | 109 (3%) |
| Komárom-Esztergom | 74 (2%) |
| Nógrád | 36 (1%) |
| Pest | 374 (12%) |
| Somogy | 85 (3%) |
| Szabolcs-Szatmár-Bereg | 205 (7%) |
| Tolna | 60 (2%) |
| Vas | 76 (2%) |
| Veszprém | 90 (3%) |
| Zala | 95 (3%) |
| All | 3147 (100%) |

**Sources:** NDGH and the authors.

**Supplementary Table 5.** Number and proportion of preventive activities provided in GP clusters in 2024.

|  | **Type of preventive service** | **Number of services provided** | **Proportion of services provided compared to all services** |
| --- | --- | --- | --- |
| 1 | Pulse oximetry test | 354 766 | 18,06% |
| 2 | Vaccination at the GP's office (administration of vaccines not included in the mandatory vaccination schedule) | 306 907 | 15,62% |
| 3 | Body composition measurement | 304 473 | 15,50% |
| 4 | 12-lead electrocardiogram (ECG) and evaluation for clinically relevant reasons | 170 240 | 8,67% |
| 5 | Screening for obstructive sleep apnea syndrome using questionnaires (STOP BANG and Berlin questionnaires, Epworth Sleepiness Scale) | 164 165 | 8,36% |
| 6 | C-reactive protein (CRP) measurement (POCT) | 51 477 | 2,62% |
| 7 | Remote blood pressure monitoring and evaluation using telemedicine | 48 304 | 2,46% |
| 8 | Assessment of risk factors for cardiovascular disease, determination of risk level in patients aged 40-65 | 47 999 | 2,44% |
| 9 | Special medical sampling and testing for infection detection (e.g., COVID, RSV, Streptococcus A) | 41 800 | 2,13% |
| 10 | Urine testing with a small laboratory machine (POCT) | 39 581 | 2,01% |
| 11 | INR measurement (POCT) | 33 229 | 1,69% |
| 12 | Use of trans-telephonic ECG in other (non-acute, clinically relevant) cases | 28 446 | 1,45% |
| 13 | Health assessment using telemedicine | 25 788 | 1,31% |
| 14 | Screening for oral cancer | 22 654 | 1,15% |
| 15 | Ankle-brachial index (ABI) measurement | 20 284 | 1,03% |
| 16 | Monitoring blood pressure percentile dynamics | 17 643 | 0,90% |
| 17 | ABPM/CBPM examination | 17 193 | 0,88% |
| 18 | Smoking dependence screening, minimal intervention | 16 685 | 0,85% |
| 19 | Stroke prevention, atrial fibrillation screening for people over 65 | 13 638 | 0,69% |
| 20 | Screening for people at risk of diabetes mellitus using the Findrisk questionnaire | 13 332 | 0,68% |
| 21 | Hearing screening with a screening audiometer | 12 736 | 0,65% |
| 22 | Depression screening with questionnaire (Beck Depression Inventory) | 12 614 | 0,64% |
| 23 | Removal of cerumen in the absence of previous ear disease | 11 814 | 0,60% |
| 24 | Professional blood sugar testing (POCT) | 11 624 | 0,59% |
| 25 | Screening for people at risk of diabetes mellitus using rapid tests | 11 274 | 0,57% |
| 26 | Alcohol dependence screening, minimal intervention | 10 505 | 0,53% |
| 27 | Microbiological sampling in cases of suspected bacterial infections (e.g., nose, throat, stool, urine, wound) | 9 796 | 0,50% |
| 28 | Medicinal inhalation treatment | 9 584 | 0,49% |
| 29 | StrepA test (POCT) | 9 530 | 0,49% |
| 30 | Dementia screening for people over 55 | 9 212 | 0,47% |
| 31 | Monitoring body weight at home using telemedicine | 8 694 | 0,44% |
| 32 | Measuring caries intensity (caries prevalence) among the population | 8 139 | 0,41% |
| 33 | Cardiovascular risk screening and care under the age of 18 | 7 922 | 0,40% |
| 34 | HbA1c% measurement (POCT) | 7 871 | 0,40% |
| 35 | Assessment of health literacy among the adult population / Health literacy parental support (in pediatric practice) | 7 305 | 0,37% |
| 36 | Sudden infant death syndrome (SIDS) prevention | 6 782 | 0,35% |
| 37 | Malnutrition risk screening in children | 4 543 | 0,23% |
| 38 | Insertion and replacement of urinary catheters | 4 480 | 0,23% |
| 39 | Use of trans-telephonic ECG in acute cardiac conditions | 4 393 | 0,22% |
| 40 | Screening for people at risk of diabetes mellitus | 4 226 | 0,22% |
| 41 | Assessing the risk of osteoporosis fractures in people over 40 | 4 108 | 0,21% |
| 42 | Blood test with a professional automatic machine | 3 858 | 0,20% |
| 43 | Monitoring and evaluation of blood sugar levels in patients' homes using telemedicine | 3 853 | 0,20% |
| 44 | ECG examination with telemetry | 3 806 | 0,19% |
| 45 | Suture and/or staple removal | 3 208 | 0,16% |
| 46 | Video consultation | 3 038 | 0,15% |
| 47 | Screening for periodontal disease, Periodontal risk screening | 2 897 | 0,15% |
| 48 | Application of the Strengths and Difficulties Questionnaire (SDQ) | 2 798 | 0,14% |
| 49 | Detection of jaw joint problems | 2 753 | 0,14% |
| 50 | Treatment and care of bronchial asthma in children under 5 years of age | 2 234 | 0,11% |
| 51 | Examination of diabetic feet | 2 210 | 0,11% |
| 52 | Preparation of dental history for ages 0-18 | 2 145 | 0,11% |
| 53 | iFOB test (POCT) | 1 896 | 0,10% |
| 54 | Following a developmental neurological examination, teaching exercises necessary for correcting discrete deviations according to the Katona method | 1 892 | 0,10% |
| 55 | D-dimer measurement (POCT) | 1 467 | 0,07% |
| 56 | Holter ECG examination | 1 184 | 0,06% |
| 57 | Early childhood development | 1 148 | 0,06% |
| 58 | Transcutaneous bilirubinometry | 1 035 | 0,05% |
| 59 | Comprehensive adolescent prevention | 1 000 | 0,05% |
| 60 | Blood sampling from fingertips in case of an epidemic | 981 | 0,05% |
| 61 | COPD screening with a questionnaire | 735 | 0,04% |
| 62 | Celiac disease screening with rapid test | 642 | 0,03% |
| 63 | Abdominal sonography | 598 | 0,03% |
| 64 | Remote monitoring – pulse oximetry examination and heart rate monitoring | 565 | 0,03% |
| 65 | Spirometry test | 519 | 0,03% |
| 66 | Troponin T measurement (POCT) | 381 | 0,02% |
| 67 | Screening for sleep apnea (OSAS) with a device | 364 | 0,02% |
| 68 | Determination of carotid-femoral pulse wave velocity (PWV) or determination of PWV using the oscillometric method, arteriography examination | 315 | 0,02% |
| 69 | Elimination and provocation tests in cases of suspected food allergy, except in cases of suspected immediate allergic reaction | 296 | 0,02% |
| 70 | Malnutrition risk screening in adults | 248 | 0,01% |
| 71 | Allergy testing with prick test | 171 | 0,01% |
| 72 | Duplex ultrasound examination of the neck veins | 167 | 0,01% |
| 73 | Duplex UH examination of peripheral blood vessels | 116 | 0,01% |
| 74 | NT-proBNP measurement (POCT) | 55 | 0,00% |
| 75 | Neck sonography | 53 | 0,00% |
| 76 | Thyroid sonography | 52 | 0,00% |

**Sources:** NDGH and the authors.

**Supplementary Table 6.** Comparison of vacant and active GP practices

|  | Vacant GP practice | | | |
| --- | --- | --- | --- | --- |
|  | Yes  (N(%) or Mean (SD)) | No  (N(%) or Mean (SD)) | Total  (N(%) or Mean (SD)) | Test |
| N | 956 (14.9%) | 5,465 (85.1%) | 6,421 (100.0%) |  |
| Practice unit type |  |  |  |  |
| Adult | 333 (34.8%) | 3,117 (57.0%) | 3,450 (53.7%) | <0.001 |
| Pediatric | 231 (24.2%) | 1,252 (22.9%) | 1,483 (23.1%) |  |
| Mixed | 392 (41.0%) | 1,096 (20.1%) | 1,488 (23.2%) |  |
| Settlement legal type |  |  |  |  |
| Village | 382 (40.0%) | 1,108 (20.3%) | 1,490 (23.2%) | <0.001 |
| small town | 63 (6.6%) | 244 (4.5%) | 307 (4.8%) |  |
| Town | 290 (30.3%) | 1,789 (32.7%) | 2,079 (32.4%) |  |
| county town | 17 (1.8%) | 183 (3.3%) | 200 (3.1%) |  |
| county capital | 105 (11.0%) | 1,046 (19.1%) | 1,151 (17.9%) |  |
| capital district | 99 (10.4%) | 1,095 (20.0%) | 1,194 (18.6%) |  |
| Settlement population | 23,973.826 (38,400.075) | 47,031.396 (52,983.231) | 43,598.436 (51,729.379) | <0.001 |
| Settlement GPs/population | 0.343 (0.284) | 0.704 (0.391) | 0.650 (0.398) | <0.001 |
| District development level |  |  |  |  |
| severely deprived | 197 (20.6%) | 459 (8.4%) | 656 (10.2%) | <0.001 |
| deprived | 81 (8.5%) | 239 (4.4%) | 320 (5.0%) |  |
| lesser developed | 238 (24.9%) | 1,077 (19.7%) | 1,315 (20.5%) |  |
| developed | 440 (46.0%) | 3,690 (67.5%) | 4,130 (64.3%) |  |
| District GPs/population | 5.067 (0.844) | 5.661 (0.835) | 5.572 (0.862) | <0.001 |
| Region |  |  |  | <0.001 |
| Southern Great Plain | 130 (13.6%) | 735 (13.4%) | 865 (13.5%) |  |
| Southern Transdanubia | 115 (12.0%) | 531 (9.7%) | 646 (10.1%) |  |
| Central Transdanubia | 124 (13.0%) | 538 (9.8%) | 662 (10.3%) |  |
| Central Hungary | 181 (18.9%) | 1,729 (31.6%) | 1,910 (29.7%) |  |
| Western Transdanubia | 93 (9.7%) | 520 (9.5%) | 613 (9.5%) |  |
| Northern Great Plain | 127 (13.3%) | 802 (14.7%) | 929 (14.5%) |  |
| Northern Hungary | 186 (19.5%) | 610 (11.2%) | 796 (12.4%) |  |

**Sources:** Authors.

**Supplementary Table 7.** Comparison of GP practices that joined and did not join the clusters

|  | GP cluster membership | | | |
| --- | --- | --- | --- | --- |
|  | Not cluster member  (N (%) or Mean (SD)) | Cluster member  (N (%) or Mean (SD)) | Total | Test |
| GP practices (N) | 2,463 (45.1%) | 3,002 (54.9%) | 5,465 (100.0%) |  |
| Sex |  |  |  |  |
| Female | 1,434 (58.2%) | 1,760 (58.6%) | 3,194 (58.4%) | 0.762 |
| Male | 1,029 (41.8%) | 1,242 (41.4%) | 2,271 (41.6%) |  |
| GP practice unit type |  |  |  |  |
| Adult | 1,382 (56.1%) | 1,735 (57.8%) | 3,117 (57.0%) | 0.015 |
| Pediatric | 545 (22.1%) | 707 (23.6%) | 1,252 (22.9%) |  |
| Mixed | 536 (21.8%) | 560 (18.7%) | 1,096 (20.1%) |  |
| Settlement legal status |  |  |  |  |
| Village | 546 (22.2%) | 562 (18.7%) | 1,108 (20.3%) | <0.001 |
| Small town | 114 (4.6%) | 130 (4.3%) | 244 (4.5%) |  |
| Town | 784 (31.8%) | 1,005 (33.5%) | 1,789 (32.7%) |  |
| County town | 67 (2.7%) | 116 (3.9%) | 183 (3.3%) |  |
| County capital | 386 (15.7%) | 660 (22.0%) | 1,046 (19.1%) |  |
| Capital district | 566 (23.0%) | 529 (17.6%) | 1,095 (20.0%) |  |
| Settlement population | 44,109.330 (51,508.470) | 49,428.814 (54,053.897) | 47,031.396 (52,983.231) | <0.001 |
| Settlement GPs/  Population | 0.725 (0.494) | 0.687 (0.278) | 0.704 (0.391) | <0.001 |
| District development level |  |  |  |  |
| Severely deprived | 246 (10.0%) | 213 (7.1%) | 459 (8.4%) | <0.001 |
| Deprived | 122 (5.0%) | 117 (3.9%) | 239 (4.4%) |  |
| Less developed | 470 (19.1%) | 607 (20.2%) | 1,077 (19.7%) |  |
| Developed | 1,625 (66.0%) | 2,065 (68.8%) | 3,690 (67.5%) |  |
| District population | 79,051.158 (55,203.470) | 91,890.016 (61,378.044) | 86,103.720 (59,017.289) | <0.001 |
| Region |  |  |  |  |
| Southern Great Plain | 336 (13.6%) | 399 (13.3%) | 735 (13.4%) | <0.001 |
| Southern Transdanubia | 223 (9.1%) | 308 (10.3%) | 531 (9.7%) |  |
| Central Transdanubia | 267 (10.8%) | 271 (9.0%) | 538 (9.8%) |  |
| Central Hungary | 842 (34.2%) | 887 (29.5%) | 1,729 (31.6%) |  |
| Western Transdanubia | 229 (9.3%) | 291 (9.7%) | 520 (9.5%) |  |
| Northern Great Plain | 322 (13.1%) | 480 (16.0%) | 802 (14.7%) |  |
| Northern Hungary | 244 (9.9%) | 366 (12.2%) | 610 (11.2%) |  |
| County |  |  |  |  |
| Borsod-Abaúj-Zemplén | 123 (5.0%) | 239 (8.0%) | 362 (6.6%) | <0.001 |
| Baranya | 77 (3.1%) | 168 (5.6%) | 245 (4.5%) |  |
| Budapest | 566 (23.0%) | 529 (17.6%) | 1,095 (20.0%) |  |
| Bács-Kiskun | 145 (5.9%) | 152 (5.1%) | 297 (5.4%) |  |
| Békés | 82 (3.3%) | 100 (3.3%) | 182 (3.3%) |  |
| Csongrád-Csanád | 109 (4.4%) | 147 (4.9%) | 256 (4.7%) |  |
| Fejér | 85 (3.5%) | 117 (3.9%) | 202 (3.7%) |  |
| Győr-Moson-Sopron | 103 (4.2%) | 128 (4.3%) | 231 (4.2%) |  |
| Heves | 192 (7.8%) | 275 (9.2%) | 467 (8.5%) |  |
| Jász-Nagykun-Szolnok | 88 (3.6%) | 103 (3.4%) | 191 (3.5%) |  |
| Komárom-Esztergom | 90 (3.7%) | 70 (2.3%) | 160 (2.9%) |  |
| Nógrád | 59 (2.4%) | 35 (1.2%) | 94 (1.7%) |  |
| Pest | 276 (11.2%) | 358 (11.9%) | 634 (11.6%) |  |
| Somogy | 86 (3.5%) | 83 (2.8%) | 169 (3.1%) |  |
| Szabolcs-Szatmár-Bereg | 104 (4.2%) | 194 (6.5%) | 298 (5.5%) |  |
| Tolna | 60 (2.4%) | 57 (1.9%) | 117 (2.1%) |  |
| Vas | 67 (2.7%) | 72 (2.4%) | 139 (2.5%) |  |
| Veszprém | 92 (3.7%) | 84 (2.8%) | 176 (3.2%) |  |
| Zala | 59 (2.4%) | 91 (3.0%) | 150 (2.7%) |  |

**Sources:** Authors.
